# Clinical Clerkship With or Without Scheduled Patient Consultations: Does It Make a Difference to Medical Students’ Experiences of Learning?

**DOI:** 10.1007/s40670-024-02160-3

**Published:** 2024-09-25

**Authors:** Christine Ilkjær, Karl-Johan Schmidt Nielsen, Louise Binow Kjær, Torben Hoffmann, Mette Krogh Christensen

**Affiliations:** 1 Department of Cardiothoracic and Vascular Surgery, Aarhus University Hospital, Aarhus, Denmark; 2 Department of Clinical Medicine, Aarhus University, Aarhus, Denmark; 3 Centre for Educational Development, Aarhus University, Trøjborgvej 82, 8000 Aarhus C, Denmark

**Keywords:** Experience-based learning, Undergraduate clinical teaching, Focus group interview, Student clinic, Student participation, CanMEDS roles

## Abstract

**Background:**

Becoming a medical expert involves leadership and professionalism, which are critical skills to learn in medical education. However, a gap exists in understanding how didactic variations in the organisation of clinical clerkships impact medical students’ opportunities to develop these skills. This study explored how clinical clerkships with or without scheduled patient consultations affect medical students’ experiences of learning leadership and professional behaviour.

**Materials and Methods:**

We conducted a qualitative, quasi-experimental study. Data were gathered through 11 end-of-clerkship focus group interviews with 87 fifth-year medical students who participated in one of two 8-week clerkships at a surgical department: a clerkship with a mentor physician or a clerkship with *scheduled patient consultations* in combination with a mentor physician. Using a constructivist lens, we analysed the focus group interview transcripts and applied grounded theory principles to the iterative coding process.

**Results:**

The analyses resulted in a descriptive framework displaying nine themes. Each theme was described as *a spectrum of meanings* that offers a nuanced understanding of how didactic variations in the organisation of clerkship impact how students ascribed meaning to the learning experiences in undergraduate medical education, focusing on learning leadership and professional behaviour.

**Discussion:**

This study investigates differences in student learning experiences when participating in a clerkship with or without scheduled patient consultation and, therefore, adds new insight into the need for a balanced, thoughtful, and didactic approach to medical education that considers both clinical exposure and co-regulated learning.

**Supplementary Information:**

The online version contains supplementary material available at 10.1007/s40670-024-02160-3.

## Introduction

Clinical clerkships are vital in preparing medical students for their future roles as doctors [1]. In a healthcare sector marked by rapid technological advancement and organisational transformation [2], there is a growing demand for generic skills among doctors, including leading evidence-based clinical decisions and recognise when to seek assistance [3], continuously striving to learn while treating patients [4], and engaging in professional development [5]. This underscores the importance of cultivating leadership and professional behaviour competencies in undergraduate medical education. However, the effectiveness of undergraduate medical education in developing these competencies comes under scrutiny, as many junior doctors encounter challenges in demonstrating them in practice [6, 7].

The significance of these competencies is underscored by the Canadian Medical Education Directions for Specialists (CanMEDS) Framework [8], a widespread guide in pre- and postgraduate medical training. This framework outlines seven roles of a competent doctor: Medical Expert, Leader, Professional, Collaborator, Communicator, Scholar, and Health Advocate. Among these roles, the Leader Role emphasises a doctor’s ability to lead oneself effectively, organise clinical work, and make clinical decisions [8]. In tandem, the Professional Role emphasises the importance of serving as a role model, respecting professional boundaries, and proactively seeking assistance when needed [8]. Despite the paramount importance of these two roles [9, 10], a notable knowledge gap exists in understanding how didactic variations within a clinical hospital department impact medical students’ opportunities to develop these competencies.

Clinical clerkships have been criticised for students mainly observing clinical work and not contributing, which is understood to impair their preparedness when transitioning into junior doctors [9]. Therefore, scheduled patient consultations or student clinics have become didactic approaches employed to transition students from observing to contributing roles in the clinical care setting [1, 11, 12]. When students are scheduled to conduct patient care under the supervision of physicians, it facilitates the development of assuming leadership and practising professionalism through contribution [1, 13–15]. However, an excessive emphasis on scheduled patient consultations during a time-limited clinical clerkship may unintentionally marginalise other valuable teaching methods, such as learning from role models and gaining proficiency in effectively navigating learning in the clinical hospital department [4, 16]. While these variations in clinical clerkship structures closely resemble the real-world scenarios junior doctors face [1, 2, 6, 7, 9], they likely represent areas in undergraduate education where students’ learning experiences diverge significantly based on how learning is organised within individual clinical hospital departments. Without a comprehensive understanding of how didactic variations within a clinical hospital department impact medical students’ learning, we cannot fully comprehend how undergraduate medical education prepares the medical doctors of tomorrow.

A conceptual cornerstone in teaching and learning the CanMEDS roles in undergraduate medical education is experience-based learning [1, 17]. According to Dornan et al., experience-based learning (ExBL) develops the capabilities and identity of a doctor, which occurs when students are supported in participation in real patient care within the educational triad of clinician, patient, and student [1]. The ExBL model implies three levels of participation:

- Observing: being present at and learning from practice without hands-on involvement.
- Rehearsing: performing tasks of practice without contributing to patient care.
- Contributing: being given the responsibility to (co-)perform tasks of practice.

Dornan and colleagues [1] described the three levels of participation as ‘rungs on a ladder’ (2019, p. 1100), with Contributing as ‘the top rung’ (2019, p. 1102). From this conceptual perspective, the study aimed to explore how *a specific didactic variation* in the meeting between clinician, patient, and student within a clinical hospital department—a clerkship *with or without* scheduled patient consultations—makes a difference to the medical students’ experiences of learning leadership and professional behaviour. The overarching purpose was to provide insights to support clinical hospital departments and medical schools in making conscious decisions to form competent medical students.

## Materials and Methods

In contrast to the commonly applied quantitative approach in experimental studies [18], we undertook a qualitative experimental inquiry to explore medical student experiences of learning opportunities in two types of clerkships in a single clinical department: one *with* and another *without* scheduled patient consultations. Thus, a quasi-experimental study [19] was designed to expose an in-depth understanding of the intervention (the scheduled patient consultations) because we assumed that when being conducted in a single clinical hospital department, all the factors influencing learning, aside from the intervention, were equal between the two study groups. This study design allowed us to explore how general (objective) structures impacted medical students’ (subjective) narratives of their learning with and without scheduled patient consultations. An interdisciplinary group of researchers conducted this study. Two (THO, CIL) researchers are clinical practitioners, supervisors, and researchers in the chosen clinical department. The other part of the group (KSN, MKC, LBK) had a scholarly background in qualitative research, medical education, and workplace learning. Consequently, the research was conducted as an iterative, reflexive practice in which our different professional qualifications offered different, and sometimes contradicting, perspectives on the data.

### Participants and Setting

Medical students participating in clinical clerkships in a single Cardiothoracic and Vascular Surgery Department were recruited consecutively from December 2017 until June 2020. Of the 92 clerkship students, 87 attended the focus group interviews (Table 1).

**Table 1:** Overview of the study design, participants, and setting

	Mentor clerkship	Intervention clerkship
Study design		
* Comparing two clinical clerkship structures (8 weeks total)*	Ad hoc work-based clinical learning: clerkship in which students were paired with a mentor physician and were free to seek learning opportunities (8 weeks)	Ad hoc work-based clinical learning (4 weeks) in combination with scheduled patient consultations in a student clinic (4 weeks)
Setting		
	One cardiothoracic and vascular surgery department	
Participants		
* Number, n*	41	46
* Women* *(n)*	(24)	(30)
* Level of education*	5th-year medical students	
* Medical school origination*	One medical school	
Data collection		
* Focus group interviews (clerkship rotation groups)*	5	6
* Duration of interviews (median; min–max)*	53 (45–59) minutes	61 (45–63) minutes

Like other surgical hospital departments [7, 20], this department had previously struggled to provide sufficient learning activities in which medical students actively experienced having a contributing role [1], because most of the clinical activity occurs in operating theatres. Therefore, we anticipated that medical students enrolled in a clerkship here were well-positioned to provide insights into how the introduction of scheduled patient consultations would affect learning experiences within an authentic clinical environment.

**Fig. 1:**
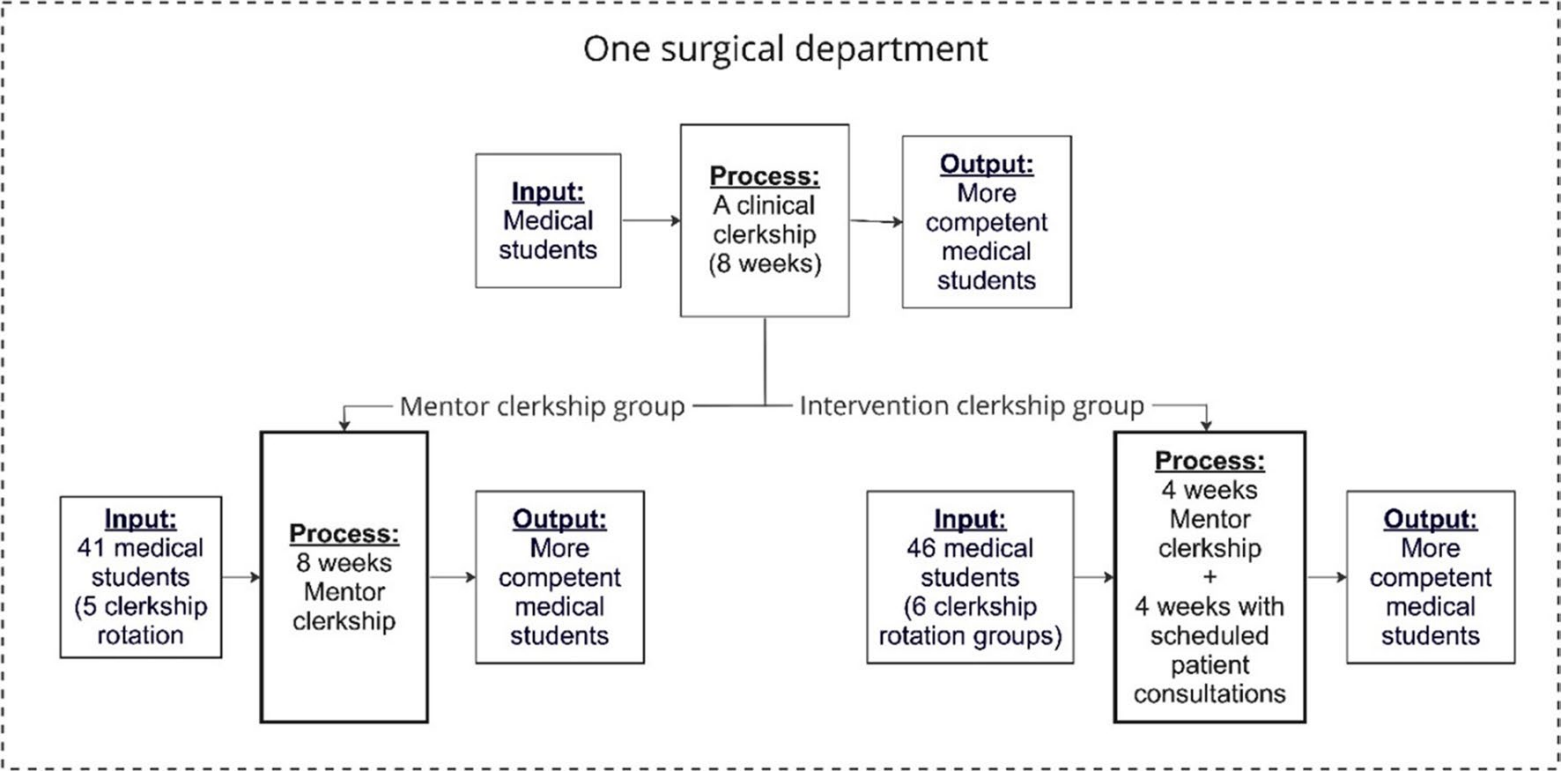
The study design

All the participating students were enrolled at the same medical school, where they took part in an undergraduate professional course focusing on the Danish version of the CanMEDS Framework [21]. At the commencement of the clerkship, students were formally introduced and given the same access to the electronic patient records system as the physicians. Students were expected to shadow a work schedule standard for junior doctors, maintain a logbook for specific tasks, and evaluate their learning experiences with the consultant in charge of the clerkship. As illustrated in Fig. 1, the 87 students were assigned to either (a) *a clerkship with a mentor physician*, which entailed an 8-week clerkship in which the students were paired with a mentor physician and were able to seek out different learning experiences when possible (e.g. participating in ward rounds or surgery); or (b) *the intervention clerkship*, in which four of the 8-week clerkship also included *scheduled patient consultations* in a student clinic.

Notably, the learning objectives were the same in both types of clerkships. The only difference between the clerkships was the scheduled patient consultation, which took place in an outpatient clinic. Here, students had formalised responsibility for patients who agreed to participate in partly unsupervised encounters with students, while the clinical teacher took a more supportive backup role [15, 22].

### Intervention: Scheduled Patient Consultations

For this study, we established a student clinic where dyads (pairs) of students held supervised *scheduled patient consultations* with newly operated cardiac surgical patients. The consultations were structured in the following way: First, the dyad of students had a pre-conference with the clinical teacher (a physician); then, the dyad of students conducted the initial conversation and clinical examination alone with the patient (sometimes also in cooperation with a cardiac nurse) with the clinical teacher on call; and finally, the clinical teacher supervised the concluding student-patient conversation. Two physicians participated as clinical teachers dedicated to the task of supervision. For newly operated cardiac surgery patients, research indicates a potential treatment benefit from closer follow-up [23, 24]. For this reason, the *scheduled patient consultations* were conducted in addition to standard postoperative care. All the patients within the intervention group volunteered to participate. Although patients in the student clinic had recently undergone cardiac surgery, our prior research has shown that patients feel safe when participating in supervised student-led consultations and patient safety was maintained [25, 26].

### Data Collection

In total, 11 focus group interviews were conducted by one of two skilled focus group moderators (MKC [*n* = 4] or LBK [*n* = 7]). Each focus group included 6–10 medical students. Using an interview guide (see ESM Appendix A), the 1-h focus group interview was structured into three sets of questions regarding learning: (A) the CanMEDS roles in general, (B) leadership, and (C) professional behaviour [27]. The initial question concerning an inquiry regarding learning across all the CanMEDS roles was meant to yield an invaluable and general impression of how the two clerkship variations—with or without scheduled patient consultations—impacted students’ overall learning experiences within the context under study. These insights were meant to form the essential groundwork for comprehending and interpreting the particular focus of our research: the students’ experiences of learning leadership and professional behaviour. The focus group participants were encouraged to share their experiences and thoughts and thus stimulated to reflect on individual, collective, and differentiated experiences. An observer external to the study was present during the interviews and wrote a summary. In 2 of the 11 focus group interviews, the interviews were conducted online due to COVID-19. The focus group interviews were recorded. Transcription was performed by a secretary external to the study.

### Data Analysis

Through a constructivist lens [19] and aligning with established recommendations in medical education research [28], we applied grounded theory principles to the inductive data analysis of the focus group interview transcripts. Since we did not employ iterative data collection and theoretical sampling to achieve saturation, the study did not qualify as a grounded theory study ‘in toto’ aiming to develop a theory [28]. Instead, we systematically processed the data using an iterative coding process, including axial coding, to develop a descriptive framework [29]. As described in ESM Appendix B, axial coding was achieved through three steps, including a systematic, iterative coding process and constant comparisons. The first step was initiated by KJC (in regular contact with CIL and MKC whenever needed) and involved open coding of the transcribed interviews, resulting in a codebook of 78 codes. After this, all authors and the clinical professor in the clinical department read all transcripts and the initial coding output before meeting to discuss and agree on the final codebook. This second step of the analysis was the process of data condensation, resulting in a codebook of nine thematic codes. After the research group had confirmed the final codebook, the final selective coding began. In this final step, MKC compared the two groups of students who had attended two types of clerkships within the same surgical department. To ensure data adequacy, we made sure that the transcripts from each focus group interview included all thematic codes, and that no new themes emerged during the analysis of the last focus group interviews. During this period, the study authors met to discuss and interpret findings when appropriate. As a result, and according to grounded theory principles applied in our data analysis process, a descriptive framework displaying *a spectrum of meanings* (see Table 2) was developed to provide a preliminary ‘theoretical explanation of the social phenomenon’ [28], in this case: medical students’ experiences of learning in a clinical clerkship with or without scheduled patient consultations. NVivo software (QRS, Doncaster, Victoria, Australia) assisted the data analysis through axial coding.

### Ethical Considerations

Ethical approval was obtained from the regional committee (1–10-72–92-19) and Aarhus University (Agr-2019–731-7573). After receiving written and verbal information, the participating medical students also gave their written consent to participate in the study.

## Results

Analysis revealed nine thematic codes, each containing descriptions of different variations—and sometimes opposite extremes—of learning experiences in the two clerkship structures in a single surgical department. The overall result was that medical students’ experience of their opportunities to learn the CanMEDS roles in general, and leadership and professional behaviour in particular, largely depends on how they feel positioned as students in the surgical department. Inspired by the work of Dornan et al. [1, 17] on experienced-based learning in clinical medical education (the ExBL model), the large variations in learning opportunities could be illustrated as rungs on a ladder. However, we found that the learning opportunities within a clinical clerkship with or without scheduled patient consultations were better described as a fluid continuum from a passive (or even absent) position to an active (or even contributing) position.

From the analysis, we developed a descriptive framework (Table 2) displaying nine themes. Each theme was described as *a spectrum of meanings* that offers a nuanced understanding of how variations in clerkship structures impact how students ascribed meaning to the learning experiences in undergraduate medical education, focusing on learning leadership and professional behaviour. For example, within the thematic code ‘Leading patient care and decision-making’, the spectrum of meaning became ‘*Challenging to navigate* vs. *Simulating*’. This illustrates that while both groups experienced having learning opportunities pertaining to leading patient care and decision-making, they ascribed different meanings to their learning. In other words, *challenging to navigate* is meaning ascribed to learning in a clerkship *without* scheduled patient consultations, whereas *simulating* is meaning ascribed to learning in a clerkship *with* scheduled patient consultations, but both meanings are embedded in the same clinical department. In this way, the spectrum of meanings describes the span of learning experiences that this particular context offers medical students. This analytical grip allowed us to construct an integrated overview of the different meanings each group of students ascribed to the clerkship structures.

**Table 2:** Descriptive framework of how students ascribed meaning to clinical clerkships with and without scheduled patient consultations

Thematic codes	Mentor clerkship	Intervention clerkship	*Spectrum of meanings*
Learning the CanMEDS roles in the clinical setting of a surgical department			
The assumed position of students	OBSERVING Students felt like observers, but possible to challenge this positioning	CONTRIBUTING Students experienced active participation irrespective of academic or professional levels	Observing vs. Contributing
Involvement and duty	SELF-ENGAGED Students were forced to seek learning opportunities and self-engage in potential opportunities (personality plays a dominant role)	SCHEDULED The scheduled tasks with a defined role make students feel involved in patient care and clear about knowing what to do	Self-engaged vs. Scheduled
Dependency on individual clinical practitioners	INDIVIDUAL The individual practitioners decide the level of student involvement in clinical work	STRUCTURAL Students’ learning practices were externally structured and mainly independent of individual practitioners	Individual vs. Structural
Learning leadership			
Leading patient care and decision-making	CHALLENGING TO NAVIGATE Often the students observed others make clinical decisions, but when leading patient care they felt the supervisor was not present/dedicated to the job	SIMULATING Frequent patient consultations trained the students to make decisions in patient care, but students felt engaged in simulated leadership	Challenging to navigate vs. Simulating
Teamwork and managing time proactively	AD HOC Students managed their own schedule, potentially with peers, in an independent and very ad hoc manner	PRE-DEFINED The pre-defined distribution of time and assignments limited integration into the larger clinical care team	Ad hoc vs. Pre-defined
The process of leadership learning	REMOTE Students observed the supervisor perform leadership, but the supervisor only sporadically supported the students in assuming the leadership role	(UN)AUTHENTIC The presence of a supervisor predicates a feeling of safety in conducting leadership, but also a feeling of undermining authentic leadership	Remote vs. (Un)authentic
Learning professional behaviour			
Professional interaction with patients	APPEARING To appear and be perceived as professional was important. However, identifying oneself as a professional was difficult when only observing others	IDENTIFYING Frequently leading patient consultations of comparable types and developing actual skills facilitated students’ confidence and identifying as a professional	Appearing vs. Identifying
Recognise and respect boundaries	SPORADICALLY Students’ professional boundaries were sporadically challenged and allowed the students to request clinical help	FREQUENTLY Students’ professional boundaries were often challenged when leading patient consultations	Sporadically vs. Frequently
Role modelling	PHYSICIANS Students met and observed a wide array of physician. The students rarely described themselves or peers as role models	PEER In-depth role modelling of a peer. Lack of observing and modelling physicians perform the specific task	Physicians vs. Peer

The three sections below describe in-depth the findings clustered in the three main themes: (1) learning the CanMEDS roles in the clinical setting of a surgical department (ESM Appendix C), (2) learning leadership (ESM Appendix D), and (3) learning professional behaviour (ESM Appendix E). When referring to quotes from the transcribed data, abbreviations such as C1 (ESM Appendix C, quote 1) are used.

### (1) Learning the CanMEDS Roles in the Clinical Setting of a Surgical Department

#### The Assumed Position of Students

The assumed position of students refers to the way in which students most frequently (and often by default) were positioned when meeting patients. In the mentor clerkship, the assumed position tended to be as an ‘observer’ and only infrequently being given the mandate of contributing to patient care (C1). In the intervention clerkship, students expressed that they were assumed to ‘contribute’ to patient care (or be the ‘helping peer’), irrespective of their academic levels, personal preferences, and prior clinical experience (C2).

#### Involvement and Duty

**Fig. 2:**
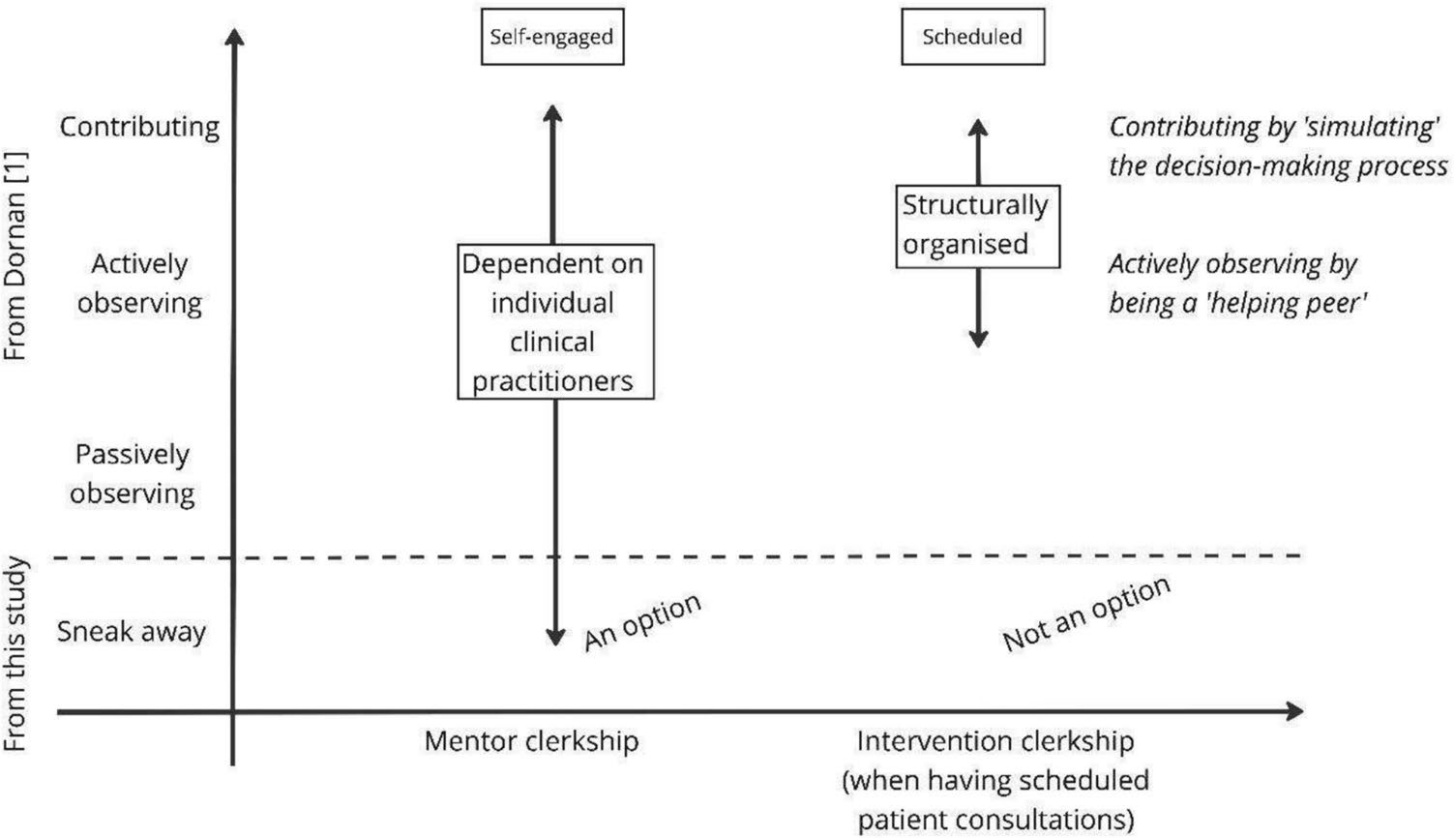
The variation of learning opportunities depending on clerkship structure. The terminology ‘observing’ and ‘contributing’ roles is inspired by Dornan et al. [1]. Texts in *italics* illustrate the meaning ascribed to learning while participating in patient care in a clerkship with scheduled patient consultations

Involvement refers to how students describe the formal structure to enable/limit their active participation in clinical work. Duty refers to whether the students were assigned clinical tasks during their clerkships and thus positioned as contributors to clinical work (see Fig. 2). Students in the mentor clerkship described how the formal structure did not clearly outline their involvement and duty within the various clinical/learning situations (C3), which prompted them to be ‘self-engaged’. In the intervention clerkship, involvement was ‘scheduled’ because patient care demanded it (C4). The students agreed they were at different levels in different subjects. Even with this sentiment, intervention clerkship students appreciated the repetitive nature of their duty, which led to a growing sense of competence in the CanMEDS Medical Expert Role, often emphasised by both the students and their clinical supervisors. The students said that because the patient consultations with newly operated patients were repetitive, they could focus on other aspects of the CanMEDS Framework, such as the CanMEDS Communicator Role (C5 + C6).

#### Dependency on Individual Clinical Practitioners

Dependency refers to the degree to which student learning experiences depend on individual practitioners (e.g. nurses or physicians) to create, support, or disrupt them. Students in the mentor clerkship stressed their dependence on ‘individual’ practitioners for their learning. Thus, forming collegial relationships was critical to being involved in tasks. While those who developed partnerships (typically through continuous mentor–mentee attachment) were usually assigned more tasks and allowed more independence (C7), those who did not build trust with individual clinical practitioners more often became observers. In the intervention clerkship, the learning situations were described as externally ‘structured’ for all aspects of student participation, and the medical students hardly referred to dependency on individual practitioners about having a contributing role (C8).

### (2) Learning Leadership

#### Leading Patient Care and Decision-Making

Leading patient care refers to experiencing how to take responsibility for patient care by observing or contributing to decision-making. Students grouped it into frequency (how often the student experienced it) and level of student engagement (vertical axis in Fig. 2). Cooperation with nurses was emphasised as an excellent opportunity for leadership training in patient consultations; in such cases, the student role felt different from their role when a doctor attended. In the mentor clerkship, students described how it was *challenging to navigate* different levels of participation, e.g. either observing physicians lead patient care or clerking with nurses and reporting to supervising physicians, which (depending on the severity of the patient care) appeared to be understood as an immense and sometimes daunting responsibility (D9). Although students frequently observed how various physicians managed to lead many types of clinical decisions (D9), they held contrasting views on whether effective learning could occur while observing clinical work in this regard (D9 + D10). Students being given/seeking the obligation to lead patient care (a contributing role [1]; see Fig. 2) could find this challenging but most often agreed with nurses and supervising physicians on how and when to discuss clinical decision-making (D9 + D11). In the intervention clerkship, students frequently led patient consultations and thus were frequently experiencing leadership in decision-making (D12). Although the level of responsibility in the student clinic was not considered to differ from situations when students in the mentor clerkship group were assigned responsibility for patient consultations (D13), the student’s views on the tasks ranged from being highly challenging in the beginning (D14) to being accessible and routine. Some students believed it was irrelevant for a peer group to conduct the patient consultation when one student could complete it alone (D15). The fact that a physician always attended the final part of the consultations in the student clinic made most students experience their decision-making and leadership of patient care as *simulating*.

#### Teamwork and Managing Time Proactively

Teamwork refers to how the students described learning through collaborating with other clinical practitioners, including dividing tasks. Managing time proactively refers to how students were expected to plan their clinical learning and proactively seek learning activities. In the mentor clerkship, cooperation with nurses was emphasised as an excellent opportunity for training teamwork (D16); in such cases, the student role felt different from their role when a doctor attended, regardless of whether the nurses also guided and supported them. Most students described a feeling of being a part of the clinical care team, mainly because they were allowed to participate in all the clinical activities. Due to having no specified duties, the students described constantly seeking learning opportunities (D17). This constant search was considered complex and frustrating for the students, impairing their motivation. Nonetheless, during the interviews, the students shared their different behaviours and strategies for managing time proactively by seeking learning experiences within the specific setting. They described a range of engagement, from extensively seeking learning experiences where they could contribute to clinical work to primarily relying on others to decide their learning journey (D18). A few students shared how they chose to sneak away and study the literature to ensure they learned ‘something’ (D19)*.* Sneaking away (see Fig. 2) from clinical work was specified as representing a vicious cycle in which the physicians could not rely on the student’s involvement in, for example, assisting with surgical procedures. Therefore, the physician did not involve the students in such duties, which made the students feel uninvolved. Overall, the students considered their learning process and outcomes to be highly dependent on their individual capability and motivation to seek learning opportunities. They managed their day-to-day activities individually and collectively using informal peer groups. Thus, teamwork and time management had an ‘ad hoc’ nature. In the intervention clerkship, both the work and whom to cooperate with for specific tasks were ‘pre-defined’ by the tight structure of the student clinic setup, leaving the focus within the team on the division of tasks. Hence, how the dyads should organise time management and teamwork within the restricted room to manoeuvre was quickly learned (D20). The students did not describe integrating into the larger clinical care team in the surgical department in the same way as those in the mentor clerkship.

#### The Process of Leadership Learning

Learning leadership refers to the student’s role in relation to the clinical supervisor when learning to assume leadership in patient care and how this role influences the learning process. All the students across the groups shared how vulnerable they felt when given responsibility for patient care because the clinical supervisor could quickly overrule their professional facade in front of the patients. In the mentor clerkship, students frequently observed physicians perform leadership, which could feel like a ‘remote’ way to learn leadership (D21). The supervisor sporadically supported students in assuming the leadership role, which could feel intimidating when not always being prepared and knowing how to handle being insecure in front of patients (D22). In the intervention clerkship, the fact that the students led the patient consultations placed them in the position of frequently experiencing a leadership role in patient care; however, it also often put them in a vulnerable position as being leaders in front of more experienced clinicians (nurse or physician) which could feel intimidating (D23).

### (3) Learning Professional Behaviour

#### Professional Interaction with Patients

Interaction with patients distinguished between frequency (how often the student experienced it) and depth (student attention level during the meeting). Professionalism was primarily viewed as a characteristic that should be directed towards the patients and, to a lesser degree, developed in conjunction with colleagues. Students across groups expressed a deep desire to appear professional and ‘fit the white coat’. Most students in the mentor clerkship described a feeling of being a part of the clinical care team. Compared to students in the intervention clerkship, students in the mentor clerkship expressed identifying themselves less as professionals and emphasised maintaining a professional ‘appearance’ towards the patients (E24 + E25) as if being professional was not a real option. In the intervention clerkship, the students described their frequent experiences with leading patient consultations as promoting a more robust sense of ‘identifying’ with a professional doctor (E26). Importantly, with a supervisor present in the patient consultation, the feeling of being recognised as a professional could potentially be challenged, but being able to convince the patients that ‘I am a capable professional who knows what I am talking about’ (E27) was part of learning professional behaviour.

#### Recognising and Respecting Boundaries

An important element of becoming a medical doctor is the ability to recognise and respect professional boundaries, that is, to know the strengths and limitations of one’s clinical knowledge and skills. Students across the groups described how being a medical student ‘legitimated’ asking other clinicians many questions without significantly impairing one’s own credibility. In the mentor clerkship, students were rarely positioned in a contributing role, and thus, their professional boundaries were tested only ‘sporadically’ (see Fig. 2). Instead, they sometimes observed other clinical practitioners recognise their own limits of expertise and ask for clinical support (E28), and this indirect way of experiencing professional boundaries was described as a reasonable learning opportunity because it resembles the everyday work of doctors. However, the sporadic and often unpredictable landscape of learning opportunities hindered the students from learning how to recognise and respect their own professional boundaries. In the intervention clerkship, boundaries were ‘frequently’ challenged, which some students described as positive. At the same time, some students considered such challenges negative because they felt they were working too far out of their comfort zones, while others experienced the supervision being too close and directive when meeting a professional boundary. Regardless of the student’s comfort levels, they all described how this frequent challenging of their boundaries helped them better identify *when* and *how* they should request clinical support (E29). Also, the close cooperation with a peer was a strength in this matter (E30).

#### Role Modelling

The students described a role model as a person others look to as an example, which can be either positive or negative in appearance. In the mentor clerkship, students appreciated observing a wide array of physicians who provided multiple examples of behaving like a professional (E31)—some were considered role models, others the opposite. Students in the intervention clerkship realised that they could actually act as role models for each other because they discovered different strengths in each other concerning how to appear as professionals and conduct clinical work (E32). On the other hand, they met fewer physicians and were less exposed to multiple examples of professional behaviour.

Further, a dedicated space, like a focus group, where students can gather and share their learning experiences within identical clerkship setups, was described as a potentially valuable resource for their educational development (F1, ESM Appendix B).

## Discussion

During undergraduate clinical clerkships, the quality of learning experiences pertaining to leadership and professional behaviour is critical for students’ development of these competencies [1, 3–5]. What sets our study design apart from previous research [6, 7, 9, 20, 30–32] is its qualitative and systematic comparison of two didactic variations of a clinical clerkship structure—one with and one without scheduled patient consultations—within a single surgical department (see Fig. 1). This study design provides a nuanced and in-depth understanding of the risks and benefits of a didactic decision requiring medical students to engage in scheduled patient consultations in a clinical clerkship. Drawing from data collected in 11 focus group interviews with 87 fifth-year medical students, our results do not assert that scheduled patient consultations are a ‘must-have’. Instead, they underscore the importance of introducing elements such as scheduled patient consultations with careful didactic considerations, as they may have unintended or even undesirable consequences on learning leadership and professional behaviour. While the preceding ‘Results’ section presents an in-depth description of our study findings, this ‘Discussion’ section delves deeper into two critical elements about the consequences of how clinical clerkships are organised: (1) the degree to which students have the opportunity to learn clinical decision-making as part of leading patient care and recognising professional boundaries, and (2) how and when students learn to engage in self-regulated learning during different types of clinical clerkships.

### Variations in Learning Opportunities Influence Students’ Learning of the Clinical Decision-Making Process

The clinical decision-making process comprises elements of assuming leadership when making decisions, as well as maintaining professionalism by realising boundaries and seeking clinical assistance when needed [3]. In Table 2, the thematic codes of ‘Leading patient care and decision-making’ and ‘Recognising and respecting boundaries’ illustrate how medical students in the two types of clerkships were exposed differently to these aspects. Our study underscores that clinical leadership and professional behaviour can be discussed and learned in a large spectrum: from an *observing* position in the mentor clerkship to an active *contributing* position in the intervention clerkship; from a highly structured learning setting such as a student clinic to a more unstructured and unpredictable landscape such as an authentic clinical hospital department. These variations in learning opportunities highlight how clerkship structures—more or less deliberately—influence students’ learning of the decision-making process, offering valuable insights for careful didactic consideration. Importantly, this study emphasises the assertion made by Dornan and colleagues, stating that both observing, rehearsing, and contributing roles [1] provide additional and valuable learning experiences.

In a grounded theory study on clinical decision-making by Kennedy et al. [3], the authors emphasise the significance of clinical training programs in facilitating prompt discussions of seeking help from supervisors in clinical decision-making among trainees. Such programs can be crucial in preventing catastrophic patient outcomes when trainees fail to seek clinical assistance effectively. Kennedy et al. [3] and subsequently similar studies [33, 34] have explored why and how *seeking help from supervisors* in postgraduate medical training is a crucial but often challenging concern. In our study, we demonstrate how implementing a student clinic in undergraduate medical training, which otherwise prioritises mentorship (see Fig. 1), influences how students learn how to *seek help from supervisors*. In the traditional mentorship-based clerkship, students often observe junior doctors demonstrating when and how to *seek help from supervisors* when given patient responsibility. In the student clinic, on the other hand, supervisors always entered the last part of the patient consultations and prompted students to go through their findings. Hence, in the student clinic, the medical students were not explicitly trained to decide *when* to seek help from supervisors, simply because they were offered help regardless of whether they decided to seek it or not.

When it comes to decision-making, it can be debated if the students’ involvement in this student clinic should be viewed as the highest level of participation in practice and the ‘top rung of the ladder’ [1], referred to as Contributing, or if it is more accurately described as Simulating. Our point here is that somewhere between Observing, Rehearsing, and Contributing, we may possibly offer Simulating as a specific term that characterises an important aspect of Contributing that allows students to train clinical skills in a way that contributes to real patient care in an authentic clinical context, but in a setting that seen from some students’ point of view resembles *simulating*—that is, acting *as if* they already have the authorisation and performative force that they need in order to be a ‘real’ doctor—when it comes to decision-making and leadership of patient care. As a result, it is worth considering whether the ExBL model [1] and its three levels of participation could unintentionally overlook the contributing potential of students participating in well-structured ‘as-if-settings’ such as scheduled patient consultations where supervisors enter the last part of the consultation to authorise decisions and leadership.

### Students’ Call for Self-Regulated Learning (SRL) Strategies

We found that the two different clerkship structures within a single clinical hospital department assign varying levels of responsibility to students in regulating their learning (see Table 2 and Fig. 2). While the *intervention clerkship* leans towards external regulation with highly structured activities guiding students’ participation in practice, students in the *mentor clerkship* perceive a substantial degree of responsibility—or internal regulation—for their own learning, even with mentorship in place. While this may not be surprising [35, 36], our research exposes how the surgical department’s didactic decisions influence how and when students learn to engage in self-regulated learning (SRL).

According to Zimmerman [37], self-regulated learners ‘focus on how they activate, alter, and sustain specific learning practices in social as well as solitary contexts’ (p. 70). Across the two clerkship types, our results highlight one essential commonality: the students call for enhanced support in formulating common and yet context-specific SRL strategies (quote F1, ESM Appendix Ba). Similarly, a recent study advocates that to support the development of students’ SRL optimally, we need to focus on facilitating and organising learners’ engagement in co-regulated learning from the start of the medical curriculum [4]. This shared sentiment transcends the boundaries of the two clerkship structures and underscores the pervasive challenges medical students face in navigating the complexities of balancing acting and adapting clinical work [37]. In a study on medical students’ clinical learning experience, Sellberg et al. [38] found that ‘the transition from learning on campus was sometimes abrupt, as the students had to switch to a more active learning role’ and that students ‘tried to adapt to their supervisors’ working situation and not to be a burden to them’. During the focus group interviews in our study, students proposed a solution to this problem by advocating for *peer focus groups during clerkship rotations dedicated to SRL strategies* in seeking learning opportunities within leadership and professional behaviour that did not depend on busy clinical teachers. This suggestion aligns seamlessly with insights from Woods et al. [20], who emphasise the advantages of context-based peer focus groups in guiding and supporting students as workplace learners. According to Woods et al., such groups have the potential to sustain motivation levels and feed valuable social comparisons [20]. Previous research has explored various methods for supporting students’ SRL, including near-peer mentorship, improved written material, or spontaneous peer-to-peer interactions [7, 30]. Although our study, in conjunction with Woods et al. [20], applied context-based peer focus groups as a data collection method, our findings suggest that the groups may also provide essential learning opportunities. Peer focus groups, consisting of students participating in the same clerkship context and at the same educational level, can potentially enhance students’ SRL capabilities across different clerkship structures. Even though this result does not allow for solid conclusions, the topic could be explored in future research to understand better whether context-based peer focus groups support students’ learning in the clinical workplace and prepare them to become competent junior doctors.

## Study Limitations

Due to the quasi-experimental design, we cannot know whether the observed differences between groups were due to actual differences, experimental manipulation, or differences between study participants. We tried to overcome these design challenges by enrolling clerkship rotation groups before and after the study intervention and enrolling many rotation groups and study participants.

This study followed grounded theory principles for data analysis, but not for data collection [28]. Usually, this would be problematic as grounded theory data collection seeks to explore a research question involving an iterative process shifting between data collection and data analysis. However, due to the quasi-experimental nature of this study [18], in which we wanted to explore the influence of an intervention on social interactions in the same clinical hospital department, we decided not to moderate the interview guide or the design of the intervention during the data collection process. We also took measures to minimise the impact of the observer effect on the results. The moderators in the focus group interviews were not from the surgical department, and the students’ anonymity was ensured.

Concerning transferability, it is essential to consider that the data were collected in one clinical hospital department. However, the fact that we concentrated the study on relatively generic skills such as leadership and professionalism allowed us to generate educational insights that may be transferred to other medical specialties.

It is important to mention that our study involved an intervention that required higher financial costs as two dedicated clinical supervisors were needed in the student clinic. Thus, the two groups being compared are unequal from an economic perspective. However, this perspective falls outside the scope of this study’s objectives.

## Implications for Medical Education

This research yields a descriptive framework (Table 2) showing that the implementation of scheduled patient consultations in a clinical clerkship, usually based on mentorship, does make a difference to medical students’ experiences of learning. Consequently, our results may provide guidance for medical schools and clinical departments when making didactic decisions about the structure of clinical clerkships, including making informed decisions on whether to include scheduled patient consultations. While participation in clinical practice is critical for medical students [1], our study shows in-depth dimensions of how different ways of structuring the students’ opportunities for participation in clinical practice can either enhance or limit their experiences of developing leadership and professional behaviour, which are important competencies in their future work as doctors [6, 7]. Results from this study support the recommendation for clinical teachers and medical schools to carefully consider how they balance the structured (but closely externally regulated) patient consultations with more accessible (but often complex to navigate) interactions with mentor physicians. An explicit ‘scope of practice’ could be for medical schools to utilise the learning opportunities in both types of clerkship structures and combine the benefits and pitfalls of the broad variations in the mentor clerkship and the more restricted room for manoeuvre in the student clinic while allowing the learners to co-regulate their learning in peer focus groups [4] and in this way distinctly nurture leadership and professional behaviour among students. Hence, the study underscores the need for a balanced, thoughtful, and didactic approach to medical education that considers both clinical exposure and co-regulated learning.

## Conclusion

Drawing from data obtained through 11 focus group interviews with 87 fifth-year medical students, this study presents a comprehensive analysis of variations in student learning in clerkship structures with and without scheduled patient consultations. The resulting descriptive framework can guide clinical teachers and medical schools in the designing of clinical education aimed at facilitating the cultivation of leadership and professional behaviour. Our results do not assert that one of the clerkship structures is superior to the other, as both offer essential but different learning opportunities. Echoing Dornan and colleagues’ concept of experience-based learning, this study visualises the way in which students’ opportunities to observe, rehearse, and contribute to real patient care in the same clinical department depend on individual as well as organisational didactic choices. The study has important implications for medical education, reminding clinical teachers and curriculum designers of the importance of balancing the fluid continuum of possible types of participation in clerkships with and without scheduled patient consultations. Concrete strategies such as peer focus groups midway through a clinical clerkship can potentially enhance the students’ awareness of their own learning in the clinical workplace and, thus, feed co-regulated learning of leadership and professional behaviour regardless of clerkship structure.

## Supplementary Information

Below is the link to the electronic supplementary material.

Supplementary file1 Appendix A-E (DOCX 30 KB)
