# Supplementary material for: Clinical Clerkship With or Without Scheduled Patient Consultations: Does It Make a Difference to Medical Students’ Experiences of Learning?
Source: Med Sci Educ. 2024 Sep 25;35(1):165–77. doi: 10.1007/s40670-024-02160-3 (PMC11933625; doi:10.1007/s40670-024-02160-3)
Supplement: Supplementary file 1 — Supplementary file1 Appendix A-E (DOCX 30 KB) [file 40670_2024_2160_MOESM1_ESM.docx]

| **APPENDIX A.** The focus group interview guide   \|  \| \| \| --- \| --- \| \| **Introduction, including a presentation of the aim of the interview, participants within the interview, and a brief introduction to the CanMEDS Roles (including short definitions)** \| \| \| **Interview questions** \| \| \| **General attitude:** \| \| \| **All CanMEDS** \| What do you think about your learning experiences and opportunities to learn the CanMEDS in this clerkship in general?  Why/why not?  How much consideration have you given to these thoughts during your clerkship?  Summary (by the interviewer) \| \| **Leadership** \| What do you think about your development within this clerkship regarding your capability to lead yourself, organise your work, and make decisions?  How much consideration have you given to these thoughts during your clerkship?  Summary (by the interviewer) \| \| **Professional behaviour** \| What do you think about your development within this clerkship regarding your capability to be a ‘role model for others’, know your professional boundaries, and seek assistance if needed?  How much consideration have you given to these thoughts during your clerkship?  Summary (by the interviewer) \| |
| --- | --- | --- | --- | --- | --- | --- | --- | --- | --- | --- | --- | --- | --- | --- |

**APPENDIX B.** The coding process

The first step involves the open coding of the transcribed interviews. This preliminary coding emphasises the systematic categorisation of statements and smaller text sections without excluding anything in advance. Hence, even though this article focuses on the CanMEDS Framework, every statement was treated as a potentially relevant piece of data. To maintain this open-minded approach towards the data and minimise cross-interview pattern recognition in this early analysis stage, the interviews were coded by alternating between the intervention and control groups. The open coding resulted in a codebook of 78 codes, in which multiple codes referenced only one or two pieces of text.

The second step of the analysis was the process of data condensation. This step aims to reach an analytically manageable number of codes without substantial data loss. Firstly, we studied the names of the codes to obtain an overview and first impression of possible cross-cutting themes. Secondly, we reduced the number of codes by thoroughly comparing the content of each code and clustering the codes that expressed similar sentiments. After two analytical iterations of this procedure, we ended up with a list of eight codes divided between three themes: *1) the context of clerkship, 2) the Leader Role, and 3) the Professional Role*. The research group then met to discuss the themes and thematic codes. At this meeting, the eight thematic codes were confirmed. An additional thematic code (‘the assumed position of the student’) was added, resulting in a list of nine thematic codes, of which three thematic codes correspond to each of the three themes. In this step, we decided to focus on the initial research questions. However, some parts of the data material revealed interesting research avenues, such as the potential positive effect on medical students participating in a peer focus group. This is exemplified in the following quote (F1):

St. 7.1: … For the record, having this focus group after two weeks in clerkship would be better for our learning outcomes. This is a forum to talk about these things and then return to the student clinic to be more aware of what we could do differently. [agreement from peer students]

Moderator: So, is this room for reflecting on learning and considering work priorities??

St. 7.1: Yes. There are some excellent suggestions for consideration, and I may not have thought of them all on my own.

The third and last step involved the actual coding. After the research group had confirmed the final codebook, the final selective coding began. In this step, the transcribed texts from the focus groups were coded based on the final codebook without generating new codes.

| **APPENDIX C.** The opportunities to learn all the CanMEDS Roles in two clinical clerkship structures | | |
| --- | --- | --- |
| **Thematic code and definitions** | **Representative transcript excerpts** | |
|  | **Mentor clerkship** | **Intervention clerkship** |
| **The assumed position of students**  Refers to how students describe their role in the student-patient-physician meeting. | Quote 1:  *‘I think the expectation is that you follow along and observe, and then you must be very outgoing to be allowed to do more. It [the task if allowed a role other than observer red.] can be challenging, depending on which doctor you pair up with’* (St.10.10). | Quote 2:  *‘Particularly in the beginning, I was a little concerned about going in there [in the student clinic red.] and shouldering the task myself. It was, however, not an option to sneak around the outpatient consultations. You had to go to that student clinic. It provided me with essential development, but in the beginning, the others had to tell me it was okay. It was highly beneficial to be pushed into* *that process’* (St. 5.7). |
| **Involvement and duty**  Refers to how the clerkship structure turns students' active participation on/off and whether the student has a specific duty in the clinical work. | Quote 3:  *‘I find it annoying that I do not have a duty; that I do not have any tasks I am responsible for in daily clinical work’* (St.2.2). | Quote 4:  *‘In the student clinic, you are allowed to do real stuff. It is great to be allowed to treat real patients and have a workday. Time seems to fly by in this setting. Other days, you might follow on the heels of a bustling surgeon and end up just observing and not saying anything – simply observing!’* (St. 9.3).  Quote 5:  *’I think in terms of it becoming a routine and the tasks being similar in nature [the patient consultations in the student clinic red.], the medical knowledge within the subject has become like knowing it by heart’* (St. 6.5)  Quote 6:  *’I believe this is the first time we have developed a routine for a particular type of patient. Typically, we encounter patients with various medical conditions, one after the other. However, here, it is consistent with the same medical condition each time (…), this has led to improved proficiency in asking specific questions, which we have not achieved previously.’ (St. 6.3)* |
| **Dependency on individual clinical practitioners**  Refers to whether the student’s learning depends on their individual clinical colleagues (e.g., physicians and nurses). | Quote 7:  *‘If you pair with the same doctor for an entire week, the doctor sizes you up and gauges your level from the very first day. Then, during the week, you are gradually entrusted with more tasks and granted increased levels of independence’* (St.10.8). | Quote 8:  *‘The fact that they [the clinical supervisors] arrive after the patient interview is really good’* (St. 8.7). |

| **APPENDIX D.** Learning leadership in the two clinical clerkship structures | | |
| --- | --- | --- |
| **Thematic code and definitions** | **Representative transcript excerpts** | |
|  | **Mentor clerkship** | **Intervention clerkship** |
| **Leading patient care and decision-making**  Refers to how students perceive leading patient care and decision-making | Quote 9:  *‘In terms of decision-making, I gain valuable insights from observing the doctors making clinical decisions, such as initiating or discontinuing medication or discharging patients. As a student, clinical decision-making can be difficult because you are often apprehensive about “Oh, will this be, okay?” and if you possess enough medical knowledge to make those clinical decisions […] However, observing the doctors helps me understand: “Argh, okay, because the patient has these symptoms, then it is okay to decide this”.’*  (St.11.5).  Quote 10:  *’It is rare [being allowed to contribute instead of observing red.], but I have encountered it a few times. However, when I weigh the little chance of having a contributing role against the certainty of gaining knowledge from studying at home, I’d prefer to manage my time effectively.’*  (St. 2.2)  Quote 11:  *‘During this clinical rotation, I believe that I have become better at making clinical decisions. I believe this progress I largely attributed to the doctors who have been supportive in granting me more responsibility. They have entrusted me with seeing patients and instructed, “Now you just do this part yourself, then you come up with a suggestion for a plan, and then we can talk about it afterwards”. I find that aspect has become more manageable for me compared to my previous clinical rotations.’*  (St. 11.2) | Quote 12:  *’We have more often been given responsibility [in the student clinic red.] because we don't have those days where you run the ass of a doctor who doesn't bother you. But I don't feel like I have more responsibility. I feel like it has been more often that I've been given a little bit more responsibility than in these either-or-experiences in a standard clinical clerkship, where you are either completely indifferent or really under pressure’ (St.6.2)*  Quote 13:  *‘I do not necessarily think we have more responsibility [in the student clinic] compared to what I have experienced in other clinical clerkship settings […here the clinical supervisor can say] “you are responsible for conducting ward rounds on two patients and reporting the plan to me before my first surgery at 9:30”. In those situations, I have just one hour to perform ward rounds on two patients, which is in stark contrast to spending 4–5 hours with three patients in the student clinic.’*  (St. 6.2).  Quote 14:  *’As a leader, you have control over things. Effective leadership requires a firm grasp of medical knowledge and a clear understanding of what is happening. However, during your first week in the student clinic, you are still learning basic skills, such as how to perform an ECG on patients and other similar tasks, which can make it difficult to achieve this level of understanding and control’* (St.8.10)  Quote 15:  *’When I’m alone with the nurse, I feel I have the leader role in the student clinic. I learn the most when I take the responsibility myself [without a peer red.]’* (St. 7.3) |
| **Teamwork and managing time proactively**  Refers to how the students experience their opportunity to assume a responsibility and the collaboration and division of tasks with peers and other employees (doctors, nurses etc.) | Quote 16:  *‘Regarding ward rounds, the nurse and I retain the established routines for pre-round preparation and post-round planning. I acknowledge their expertise, and they share essential information with me, enabling me to make informed decisions. [When we are in the presence of the patient], they subtly guide me on standard procedures.’* (St. 10.5).  Quote 17:  *’We have had a lot of time for our tasks, so our planning has concentrated on how we learn best during the day. For example, we can choose to attend surgery in the morning.’* (St. 11.7)  Quote 18:  *‘I believe the general expectation is that students observe and follow along, and if you want to take on a more active role, you need to be assertive. However, the level of difficulty in achieving this varies depending on the doctor you follow along. Among those I have accompanied, very few have proactively offered opportunities for involvement. It has largely been up to the students themselves to proactively seek out learning experiences. I believe our individual experiences in this regard have been quite diverse.’* **(**St. 10.10)  Quote 19:  *’For me, this clinical stay has been very much like “okay, I am responsible for my own learning”, and therefore I have made assessment of standing 5 hours in the operating theatre, which I don’t learn anything from. So instead, I have found a spot to study in my books. I have done this many days because I believe that is where I learn most. In the end, there is an exam I must pass, and those subjects does not include my time in the operating room’* (St. 1.2) | Quote 20:  *‘So, before we started the consultation […], we had these 5 minutes; I take those two tasks, and you [the peer] do that, and then afterwards, I can do that while you [the peer] prepare that. You quickly learned to collectively organise’* (St. 5.1). |
| **The process of leadership learning**  Refers to how students describe their leadership training while clinical supervisors are present | Quote 21:  *’Well, it is disheartening to feel like you are just air, not seen or spoken to at all. And vice versa, you can really grow from learning some medical knowledge or getting constructive feedback on your medical knowledge or professional appearance’* (St. 10.10)  Quote 22:  ‘*In the clinical situation, when I auscultate a patient's heart and the doctor kindly asks me,” So, what do you hear?” Then I feel like I am being tested in front of the patient because I am uncertain about what I hear. And I don’t want to say “I hear a holosystolic murmur” in front of the patient when I am not entirely secure in my assessment’* (St. 1.1). | Quote 23:  *‘I think that you doubt your own knowledge a bit more when there is a person that knows more than you. And then you are like, okay, I could potentially answer this, but you can answer instead because you probably have a better way to answer this question than I do. A better way of communicating’* (St. 8.3). |

| **APPENDIX E.** Learning professional behaviour in the two clinical clerkship structures | | |
| --- | --- | --- |
| **Thematic code and definitions** | **Representative transcript excerpts** | |
|  | **Mentor clerkship** | **Intervention clerkship** |
| **Professional interaction with patients**  The students’ reflections on their manner and charisma in contact with patients | Quote 24:  *‘I think one thinks about how one appears when conducting rounds or something, where you must be respectful towards the patient. Especially here, where every patient is dealing with a potentially life-threatening condition. Given these circumstances, I often think about how I appear’* (St. 10.7).  Quote 25:  *’I believe that as a medical student, you hold a crucial role because you represent the entire medical profession. It is important to recognize that patients may not always perceive you as being at the bottom of the hierarchy’* (St. 10.10) | Quote 26:  *‘I have been well-prepared to address the primary questions they [the patients] tend to ask. That has made me feel more professional.’*  (St. 5.3).  Quote 27:  *‘In other words, one's professional role is also challenged when they [the clinical supervisors red.] enter the room because the patient wants to look at them because “the doctor arrived”. Nonetheless, being able to convince them [the patients red.] that I am a capable professional who knows what I am talking about has been valuable to learn. In other words, I think that part of being professional has been beneficial to practice’* (St. 7.4) |
| **Recognise and respect boundaries**  The students' experiences of having responsibility for patient care and perceived decision-making competence. | Quote 28:  *‘I have seen even the most experienced consultants saying, “Do you know what, that is not my medical expertise. I cannot answer you and know it's correct, so you need to ask people in another medical speciality”. From these situations, I have learned that even though you have graduated and are a consultant, you don’t need to know everything.’*  (St. 11.7). | Quote 29:  *‘I also think that the student clinic is a really good room for learning […] because it is a room where you are allowed to ask. The supervisors don't get mad at being asked. You know that in advance, so it is also a little easier than going and knocking on an office and asking.’* (St. 5.7)  Quote 30:  *‘I hardly feel like a failure when I consult a peer student compared to asking the physicians’* (St 4.3). |
| **Role modelling**  How the students understood each other, the physicians, and the nurses as role models. | Quote 31:  *‘In this surgical department, there are some very engaged physicians dedicated to their profession. They spend an enormous time at work. I think it is cool to see someone being so dedicated to their job. They are good role models’* (St. 10.10). | Quote 32:  *‘It has been good for me to work in pairs because [student X] has some excellent competencies, as a future physician, that I don’t. We are different, but we work well together, and I have learned a lot from our collaboration’* (St. 4.4). |
